# Piperine Enhances the Antioxidant and Anti-Inflammatory Activities of Thymoquinone against Microcystin-LR-Induced Hepatotoxicity and Neurotoxicity in Mice

**DOI:** 10.1155/2019/1309175

**Published:** 2019-04-16

**Authors:** Mohamed M. Abdel-Daim, Amany A. Sayed, Ahmed Abdeen, Lotfi Aleya, Daoud Ali, Abdullah A. Alkahtane, Saud Alarifi, Saad Alkahtani

**Affiliations:** ^1^Pharmacology Department, Faculty of Veterinary Medicine, Suez Canal University, Ismailia 41522, Egypt; ^2^Zoology Department, Faculty of Science, Cairo University, Giza 12613, Egypt; ^3^Department of Forensic Medicine and Toxicology, Faculty of Veterinary Medicine, Benha University, Toukh 13736, Egypt; ^4^Bourgogne Franche-Comté University, Chrono-Environment Laboratory, UMR CNRS 6249, 25030 Besançon Cedex, France; ^5^Department of Zoology, Science College, King Saud University, Riyadh, Saudi Arabia

## Abstract

Microcystin- (MC-) LR is the most frequent cyanotoxin produced by *Microcystis aeruginosa* cyanobacteria in the contaminated freshwater environment. MC represents a health hazard to humans and animals. Therefore, the present study was designed to evaluate the potential ameliorative effect of thymoquinone (TQ) and/or piperine (PP) against MC toxicity in mice. Fifty-six mice were randomly divided into seven experimental groups. Group I is the normal control that received distilled water for 21 days; Group II (TQ) was treated with TQ (10 mg/kg, i.p) for 21 days; Group III (PP) was treated with PP (25 mg/kg, i.p) for 21 days; Group IV (MC) was treated with MC (10 *μ*g/kg, i.p) for 14 days and served as the toxic control; and Groups V, VI, and VII received TQ and/or PP 7 days prior to MC and continued for 14 days with MC. The results revealed that MC elicited hepatotoxicity and neurotoxicity which was evident due to the significant elevation of serum AST, ALT, *γ*GT, ALP, LDH, IL-1*β*, IL-6, and TNF-*α* levels. Furthermore, MC markedly increased MDA and NO contents along with reduction of GSH, SOD, CAT, and GSH-Px in liver and brain tissues. The electron transport chain may be a possible target for MC. TQ and/or PP ameliorated the MC-mediated oxidative damage in the liver and brain which might be attributed to their antioxidant properties. However, the concurrent treatment of TQ and PP showed the best regimen as a result of the PP-enhanced bioavailability of TQ.

## 1. Introduction

Microcystin- (MC-) LR is the most abundant cyanotoxin released by *Microcystis aeruginosa* cyanobacteria (blue-green algae) into freshwater bodies as a result of extensive anthropogenic eutrophication [1]. MC, which is known to withstand higher temperatures (up to 300°C) increasing its persistence in the aquatic ecosystem, together with excessive production lead to the build-up of high concentrations in the environment offering potential sources of exposure for humans, animals, fish, and birds, mainly through consumption of contaminated seafood, vegetables, and drinking water [2, 3]. MC is described to be a potent hepato- and neurotoxin [2, 4–6]. MC poisoning has been recently reported in many countries including China [7], Egypt [8], Japan [9], Poland [10], and Brazil [11, 12]. Additionally, the MC-prolonged exposure promotes oncogenesis in humans [13], together with the abovementioned; MC has become a great global health concern.

MC is known to exert its hepatotoxicity and neurotoxicity via the inhibition of phosphatase enzymes [4]. Through the biliary system, MC reaches the hepatocytes, wherein it has high affinity with the serine/threonine-containing enzyme phosphatases causing hyperphosphorylation and enzyme dysfunction. These events disrupt the signaling pathways and cytoskeleton integrity; thus, MC-induced hepatotoxicity occurs [4, 14, 15]. Independent to these mechanisms, several lines of recent evidence reported the involvement of oxidative stress in MC-induced toxicities through the overproduction of reactive oxygen species (ROS) and suppression of the cellular antioxidant system [6, 16]. However, the exact mechanisms underlying the MC-induced toxicity remain unclear. Therefore, exploring new mechanistic insights, as well as new antioxidative agents, which protects against MC-inflicted toxicities would be of great impact on animal and human health.

Recently, attention has been paid to the role of medicinal plants as a source of natural antioxidants. Thymoquinone (TQ) is the main constituent of *Nigella sativa* (black seed) with antioxidant, anti-inflammatory, and anticarcinogenic activities. Several reports have documented the protective effect of TQ against the oxidative damage inflicted by paracetamol [17] and lead [18] in the liver; TQ could also alleviate the inflammation associated with Alzheimer's disease [19].

Piperine (PP) is the bioactive alkaloid ingredient of black pepper (*Piper nigrum*). PP has various pharmacological potencies including antioxidant and anti-inflammatory actions [20, 21]. Recently, coadministration of plant extracts has gained great attention, whereas using two or more components may enhance their clinical efficacy [22]. Lambert et al. [23] have demonstrated that the bioavailability of (-)-epigallocatechin-3-gallate was increased by 1.7-fold when it was coadministrated with PP in a mouse model. Despite the proven antioxidant activity of TQ, it has poor bioavailability due to its lower water solubility, poor absorption when given orally, and rapid elimination from plasma [24]. Accordingly, we hypothesize that PP may potentially enhance the antioxidant and anti-inflammatory efficacy of TQ by increasing its bioavailability. The present study was designed to comparatively evaluate the therapeutic potential of TQ, PP, and their combination against the MC-induced oxidative damage in hepatic and brain tissue. Liver function tests, proinflammatory cytokines, and oxidative status markers were determined.

## 2. Materials and Methods

### 2.1. Chemicals

Microcystin- (MC-) LR, thymoquinone (TQ), and piperine (PP) were procured from Sigma-Aldrich (Saint Louis, MO, USA). All kits used for biochemical and oxidant/antioxidant markers analyses were purchased from the Laboratory Biodiagnostics Company (Cairo, Egypt) except the lactate dehydrogenase (LDH) enzyme kit (Randox Laboratories Ltd., UK). Interleukin-1*β* (IL-1*β*) and Interleukin-6 (IL-6) were obtained from Glory Science Co. Ltd. (Del Rio, TX, USA) and tumor necrosis factor-*α* (TNF-*α*) was purchased from BioSource International Inc. (Camarillo, CA, USA) to assess the inflammatory responses.

### 2.2. Animals

Adult male albino mice (*Mus musculus*) weighing 25-30 g were used in the present study. They were housed and maintained in controlled conditions at 25 ± 2°C with a 12 : 12 light-dark cycle. Food and water were given *ad libitum*. Before commencement of the experiment, all animals were acclimatized to the laboratory conditions for 7 days.

The experimental protocols and procedures used in this study were approved by the Cairo University, Faculty of Science, Institutional Animal Care and Use Committee, Egypt (CU/I/F/39/18). All the experimental procedures were carried out in accordance with international guidelines for the care and use of laboratory animals.

### 2.3. Experimental Protocol

Fifty-six mice were equally divided into seven experimental groups. Group I (control): mice received distilled water as a vehicle control only for 21 days. Group II (TQ): mice received thymoquinone (10 mg/kg daily, i.p; Talib (2017)) for 21 days. Group III (PP): mice received piperine (25 mg/kg daily, i.p; Talib (2017)) for 21 days. Group IV (MC): mice received microcystin-LR (10 *μ*g/kg daily, i.p) for 14 days and then were administered distilled water daily for the subsequent 7 days and served as the MC-intoxicated group [25]. Group V (MC-TQ): mice received thymoquinone only for 7 days and then were treated with TQ and MC concurrently for the subsequent 14 days. Group VI (MC-PP): mice received piperine only for 7 days and then were treated with PP and MC concurrently for the subsequent14 days. Group VII (MC-TQ-PP): mice were coadministered with TQ and PP for 7 days and then treated with TQ, PP, and MC concurrently for the subsequent 14 days.

### 2.4. Blood and Tissue Sampling

After 24 h from the last treatment, all groups were euthanized under sodium pentobarbital overdose and blood samples were collected via heart puncture. The blood sample was centrifuged and the serum collected and stored at −20°C for further determination of liver function tests and proinflammatory cytokines (IL-1*β*, IL-6, and TNF-*α*). The liver and brain were rapidly excised, perfused in physiological saline, and homogenized in ice-cold 0.2 M Tris-HCl buffer (pH 7.4) followed by cooled centrifugation (4°C) at 10000 x*g*. Then, the resultant supernatant was collected and stored at -80°C for determination of tissue oxidative cascade markers.

### 2.5. Determination of Hepatic-Specific Markers

Serum levels of aspartate aminotransferase (AST) and alanine aminotransferase (ALT) were assessed according to Reitman and Frankel [26], while that of alkaline phosphatase (ALP) was determined according to Tietz et al. [27]. Furthermore, gamma-glutamyl transferase (*γ*GT) and lactate dehydrogenase (LDH) activities were evaluated according to Szasz [28] and Babson and Babson [29], respectively.

### 2.6. Determination of Proinflammatory Cytokines

IL-1*β*, IL-6, and TNF-*α* proinflammatory markers were assayed using commercial ELISA kits according to the manufacturer's instructions, and the absorbance values were measured using an automated ELISA reader at 450 nm.

### 2.7. Evaluation of Liver and Brain Oxidative/Antioxidative Markers

The oxidant/antioxidant status was evaluated in the previously prepared liver and brain tissue homogenates through determination of malondialdehyde (MDA) [30] and nitric oxide (NO) [31] concentrations. In addition, the content of antioxidant molecules including reduced glutathione (GSH), superoxide dismutase (SOD), catalase (CAT), and glutathione peroxidase (GSH-Px) was also determined in the liver and brain according to Beutler et al. [32], Nishikimi et al. [33], Aebi [34], and Paglia and Valentine [35], respectively.

### 2.8. Statistical Analysis

All results were presented as the mean ± SE from eight mice per group. The results were analyzed statistically by using a statistical program (version 21.0; SPSS Inc., Chicago, IL, USA). First, all data were tested for normality (Shapiro-Wilk's test) and homogeneity (Levene's test) as well. Then, one-way analysis of variance (ANOVA) was used to analyze the relationship between multiple groups, followed by Tukey's test under a probability of 0.05.

## 3. Results

### 3.1. Effect of TQ and/or PP Treatment on Hepatic Markers

**Figure 1:**
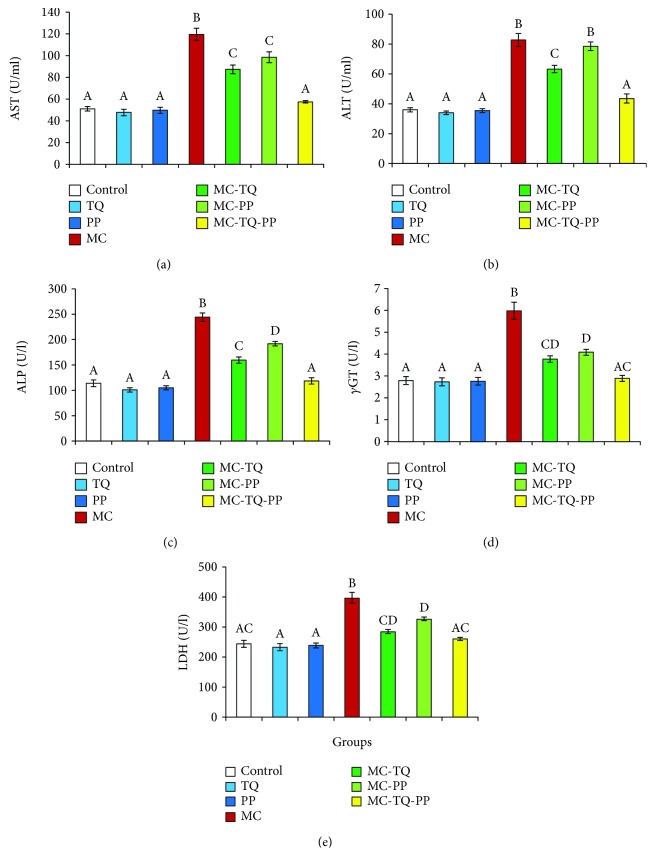
Ameliorative effect of thymoquinone (TQ) and/or piperine (PP) against microcystin- (MC-) LR-induced hepatotoxicity in mice. (a) AST; (b) ALT; (c) ALP; (d) *γ*GT; (e) LDH. Values are expressed as mean ± SE. Columns carrying different letters are considered significantly different (*P* < 0.05).

As depicted in Figure 1, MC caused hepatotoxicity indicated by significant (*P* < 0.05) elevations of serum AST, ALT, ALP, *γ*GT, and LDH levels of 234.34%, 229.74%, 214.33%, 214.15%, and 162.17%, respectively, when compared to the normal controls. On the other hand, the treatment of MC-intoxicated mice with TQ or PP exerted significant improvements in these hepatic markers denoted by marked (*P* < 0.05) decreases in AST, ALT, ALP, *γ*GT, and LDH levels (MC-TQ: 73.11%, 76.42%, 65.37%, 63.03%, and 71.72% and MC-PP: 82.40%, 94.85%, 78.54%, 68.49%, and 82.33%, respectively) in comparison to MC-treated animals (Figure 1). Obviously, when TQ and PP were given in combination, they could significantly (*P* < 0.05) restore all previous parameters close to the normal values than their individual treatments to reach 48.01%, 52.56%, 48.56%, 48.31%, and 65.74%, respectively, when compared to the MC values.

### 3.2. Effect of the TQ and/or PP Treatment on Inflammatory Reaction

**Figure 2:**
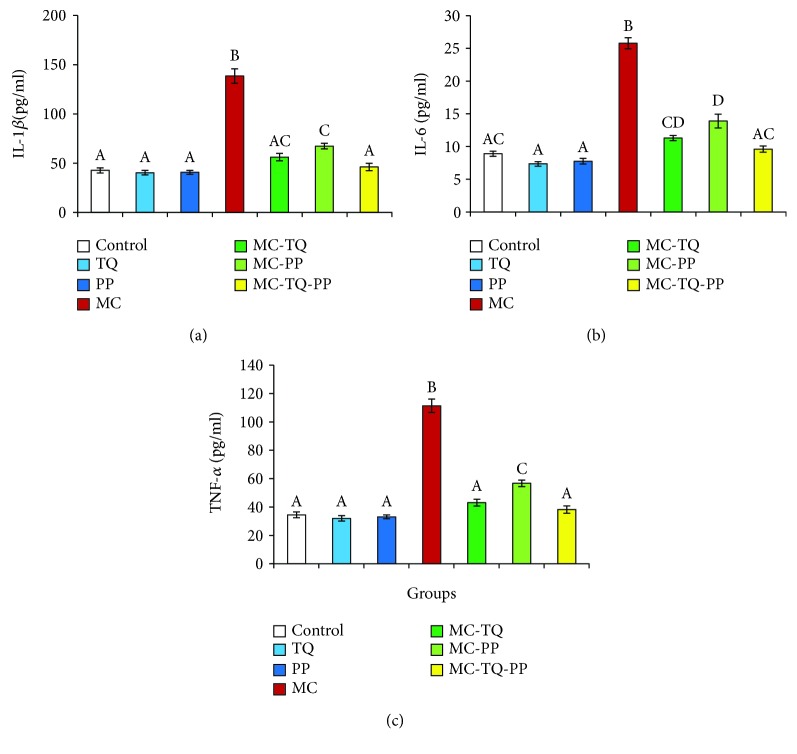
Ameliorative effect of thymoquinone (TQ) and/or piperine (PP) against microcystin- (MC-) LR-induced inflammation in mice. (a) IL-1*β*; (b) IL-6; (c) TNF-*α*. Values are expressed as mean ± SE. Columns carrying different letters are considered significantly different (*P* < 0.05).

Serum concentrations of inflammatory markers (IL-1*β*, IL-6, and TNF-*α*) were dramatically (*P* < 0.05) higher in the MC-treated mice than in the saline-treated ones (323.99%, 289.12%, and 323.00%, respectively) as shown in Figure 2. However, treatment of MC-intoxicated mice with TQ or PP significantly (*P* < 0.05) lowered serum IL-1*β*, IL-6, and TNF-*α* concentrations (MC-TQ: 40.52%, 43.85%, and 38.68% and MC-PP: 48.85%, 53.95%, and 50.97%, respectively) in comparison to the MC group. Interestingly, the MC-TQ-PP group showed marked decreases in the measured inflammatory cytokines to 33.29%, 37.26%, and 34.37%, respectively, relative to MC alone suggesting that the anti-inflammatory effects of the TQ-and-PP-combined therapy were more effective than those of each individual treatment (Figure 2).

### 3.3. Effect of the TQ and/or PP Treatment on Hepatic Oxidant/Antioxidant Status

**Figure 3:**
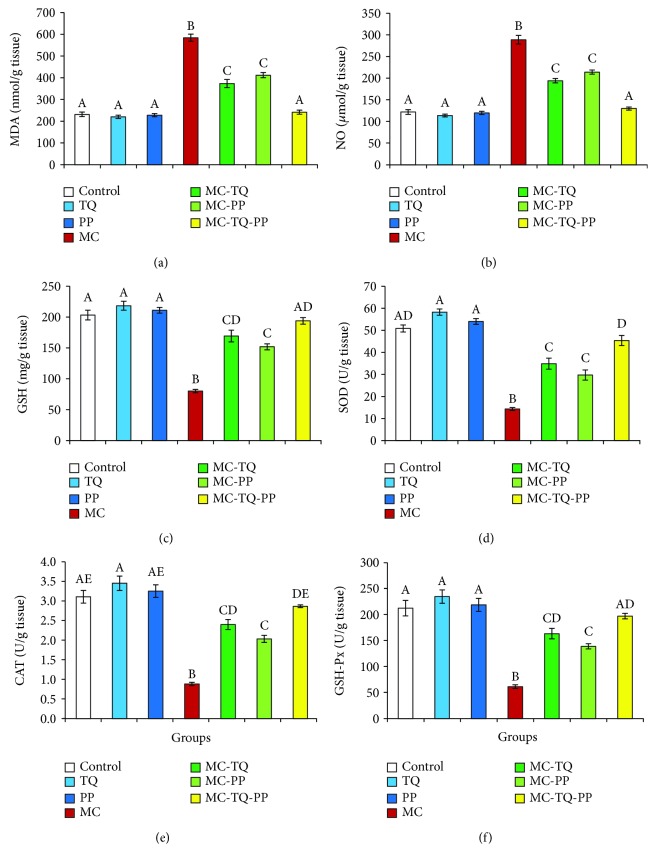
Ameliorative effect of thymoquinone (TQ) and/or piperine (PP) against microcystin- (MC-) LR-induced oxidative damage in the mouse liver. (a) MDA; (b) NO; (c) GSH; (d) SOD; (e) CAT; (f) GSH-Px. Values are expressed as mean ± SE. Columns carrying different letters are considered significantly different (*P* < 0.05).

As shown in Figure 3, MC evoked oxidative damage in the liver that was evident by dramatic (*P* < 0.05) increases in MDA and NO concentrations (252.83% and 236.52%, respectively) and decreases in GSH, SOD, CAT, and GSH-Px contents (39.42%, 28.17%, 28.42%, and 28.85%, respectively) in comparison to the corresponding control group. Alternatively, treatment with TQ, PP, or both attenuated the MC-induced hepatooxidative damage indicated by marked (*P* < 0.05) reductions in MDA (63.90%, 70.48%, and 41.37%, respectively) and NO (67.26%, 74.10%, 45.12%, respectively) when compared with MC-intoxicated animals. Further, it was observed that each of the TQ and PP was able to significantly (*P* < 0.05) elevate the MC-reduced GSH levels and SOD, CAT, and GSH-Px activities (MC-TQ: 211.18%, 243.57%, 271.96%, and 266.34% and MC-PP: 189.57%, 207.60%, 230.14%, and 226.48%, respectively) as compared with the MC values. In addition, the cotreatment of TQ and PP boosted the antioxidant molecules more profoundly than each single regimen seen by marked (*P* < 0.05) increases in the hepatic GSH, SOD, CAT, and GSH-Px contents by 242.09%, 316.91%, 324.46%, and 321.52%, respectively, relative to that of the MC group.

### 3.4. Effect of the TQ and/or PP Treatment on Brain Oxidant/Antioxidant Status

**Figure 4:**
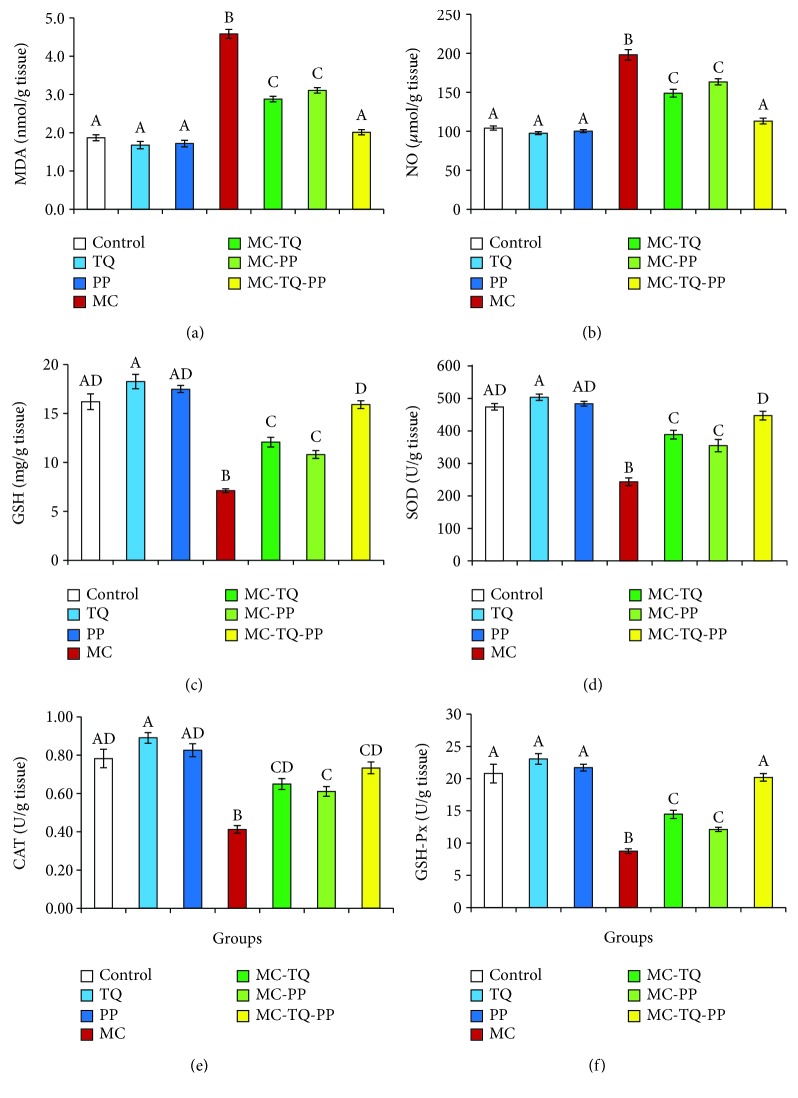
Ameliorative effect of thymoquinone (TQ) and/or piperine (PP) against microcystin- (MC-) LR-induced oxidative damage in the mouse brain. (a) MDA; (b) NO; (c) GSH; (d) SOD; (e) CAT; (f) GSH-Px. Values are expressed as mean ± SE. Columns carrying different letters are considered significantly different (*P* < 0.05).

Figure 4 displays that MC provoked drastic (*P* < 0.05) increases in brain MDA and NO concentrations (245.15% and 190.04%, respectively) as compared with the control group. Our results also revealed a reduced antioxidant capacity depicted by marked (*P* < 0.05) decreases in the GSH level and SOD, CAT, and GSH-Px activities (43.85%, 51.33%, 52.76%, and 41.97%, respectively) in the brain tissue of MC-intoxicated mice. Nevertheless, treatment with TQ or PP was effective in diminishing oxidative damage induced by MC, wherein TQ could obviously (*P* < 0.05) lower the MDA and NO levels (62.77% and 75.11%, respectively) accompanied by significant (*P* < 0.05) increases in brain GSH, SOD, CAT, and GSH-Px contents (169.90%, 159.73%, 157.73%, 165.70%, respectively) in comparison to toxic controls. In the same line, PP could markedly (*P* < 0.05) decrease the levels of the MC-increased MDA and NO to 67.72% and 82.44%, respectively, and elevate the contents of GSH, SOD, CAT, and GSH-Px in brain tissue (152.02%, 145.80%, 148.02%, and 138.69%, respectively) relative to MC controls. Notably, combined therapy showed better improvement in the antioxidant status of brain tissue of MC-treated mice proven by significant (*P* < 0.05) reductions in MDA and NO levels (43.81% and 57.15%, respectively) and upregulated GSH level and SOD, CAT and GSH-Px activities (223.82%, 183.77%, 177.62%, and 231.15%, respectively) in comparison with the initial MC values (Figure 4).

## 4. Discussion

Extensive anthropogenic eutrophication of water bodies leads to growth of cyanobacteria and consequently the production of high amounts of microcystin- (MC-) LR which is a potent environmental hazard to human, animal, fish, and bird health. Consumption of contaminated food and drinking water is the main source of exposure [1]. It is well known that the liver is the primary target of MC intoxication [4, 15]. In the same line, our study revealed severe hepatotoxicity after MC treatment indicated by marked elevations of serum AST, ALT, and ALP levels. These findings support the data obtained by previous reports [1, 36, 37] which studied the change in liver function during MC insult. Previous studies have attributed the MC-induced hepatotoxicity to the high affinity of MC to form strong covalent bonds with hepatic serine/threonine-specific protein phosphatases which potently inhibit their activities [14]. These events initiate a cascade of cellular dysfunction including disturbing cellular signaling and loss of cytoskeletal integrity due to protein hyperphosphorylation, ending in the leakage of liver marker enzymes into the bloodstream [4, 5, 15, 38].

Moreover, a growing body of evidence suggests the involvement of oxidative stress in MC-induced hepatotoxicity [1, 6, 14, 37, 39]. Besides the hepatotoxicity, MC has the ability to cross the blood-brain barrier [40] and cause neurotoxicity via possible mechanisms of phosphatase inhibition and oxidative damage [2, 5, 41]. In the biological system, oxidative stress comprises a sequence of events which involves the excessive production of ROS (O_2_^•−^ (superoxide anions), OH^•^ (hydroxyl radicals), H_2_O_2_ (hydrogen peroxide), and NO (nitric oxide)) that overwhelms the cellular antioxidant defense mechanisms. These events cause several deleterious effects on cellular macromolecules such as lipid peroxidation, mitochondrial dysfunction, ATP reduction, protein oxidation, and DNA damage [38, 42, 43]. OH^•^ is the most harmful ROS that directly attacks the lipid content of the cellular membranes causing lipid peroxidation manifested by increased production of MDA that also has the affinity to bind to other cell molecules which worsens the situation. Among cellular antioxidants, GSH, CAT, and GSH-Px are required for hydrolysis of H_2_O_2_ into H_2_O. However, depletion of such antioxidants makes the hydrolysis reaction shift to Fenton's reaction, where large amounts of OH^•^ are formed from H_2_O_2_ [43]. Consistently, this study suggests the implication of oxidative stress during MC intoxication which was observed by significant increases in MDA and NO levels along with the depletion of cellular antioxidants in liver and brain tissues. In addition, in the current study, loss of hepatocyte membrane integrity due to MC-evoked lipid peroxidation may be another cause of the efflux of transaminases into the bloodstream [38, 42].

Furthermore, MC has been reported to accumulate in the liver as a part of its detoxification process, wherein it forms a conjugate with hepatic GSH, namely, the MC-GSH complex [14]. That leads to exhaustion of the available GSH which is required for the recycling of GSH-Px during the hydrolysis reaction of H_2_O_2_ into H_2_O [44]. Our data also showed decreases in SOD activities which are the main O_2_^•−^ scavenger. Therefore, the accumulated O_2_^•−^ spontaneously reacts with the excess NO and forms the peroxynitrite radical (ONOO^−^) which causes protein nitration and dysfunction and depletion of GSH [25, 44], recently called nitrosative stress.

*γ*GT is described as a sensitive marker for oxidative stress [45]. *γ*GT is a membranous protein expressed in many cell types, particularly in the liver. It is expressed mainly in the apical plasma membrane facing the extracellular space [46], where it functions as a GSH recycler [47]. Therefore, we suppose that the increased serum level of *γ*GT and decreased tissue concentrations of GSH may be attributed to the loss of membrane integrity and release of *γ*GT into the bloodstream as a result of MC-induced lipid peroxidation along with reduced GSH recycling. Our data confirm the studies which reported an inverse correlation between *γ*GT and antioxidants [45]. The current study also reported a significant increase in the serum LDH level. Since the cell integrity is lost due to lipid peroxidation, LDH is released extracellularly and increases its serum level [48]. It is well known that when there is a reduction in ATP production along the electron transport chain during the aerobic glycolysis, the reaction shifts to anaerobic glycolysis where lactates and LDH are increased [25, 48]. Our group has previously demonstrated an increased serum LDH in a fipronil-targeted electron transport chain [38]. Therefore, we suppose that the electron transport chain might be a possible target for MC.

The present study reported marked increases in serum *γ*GT and tissue NO, besides the intracellular accumulation of MC. All of these events played a central role in GSH exhaustion and subsequent reduction in GSH-Px, SOD, and CAT activities. Taken altogether, our data confirm the involvement of oxidative damage in MC-induced toxicity [1, 2, 6, 14, 37, 39, 41].

It was well known that excessive ROS production and oxidative stress initiate an intracellular cascade signaling that enhances the expression of the proinflammatory gene as well as the production of inflammatory cytokines [49]. Along with these, the present investigation revealed increases in serum IL-1*β*, IL-6, and TNF-*α* which might happen in response to MC-induced oxidative stress in the liver and brain tissue. The ongoing findings are in agreement with Ahmad et al. [1], Abd AL Majeed et al. [50], and Lone et al. [25]. The current data also emphasize our previous reports which demonstrated a triggered inflammatory reaction in response to oxidative damage induced by piroxicam [51], acetaminophen [52], fipronil insecticide [38], and cadmium [42] insults. Basically, the oxidative insult besides the proinflammatory cytokine, recorded in the present result, increases the vulnerability of the brain to various neurodegenerative diseases.

TQ is described to have anti-inflammatory and antioxidant properties exerted by its ROS scavenging and antioxidant upregulation activities [17, 18]. PP is also reported to have the same pharmacological actions [20, 21]. In the present investigation, treatment with TQ or PP attenuated the MC-induced hepatotoxicity and neurotoxicity indicated by significant reduction of the serum biochemical parameters and proinflammatory cytokines along with improvement in the oxidative/antioxidative state. In fact, the chemical structure of TQ and PP is the key of their antioxidant activity. Their chemical structures contain a phenol ring, double bonds, and hydrogen atom (H^+^) allowing free delocalization of electrons and H^+^ donation by which scavenging and neutralization of ROS, NO, and accumulated MC take place and eventually attenuate the oxidative damage [52]. Supplementation with external ROS scavenging agents increases the availability of the cellular unconjugated GSH promoting the cellular antioxidant capacity. In another cohort study, dietary supplementation of antioxidants has shown a negative correlation with serum *γ*GT suggesting the improvement of cellular oxidative status [45].

**Figure 5:**
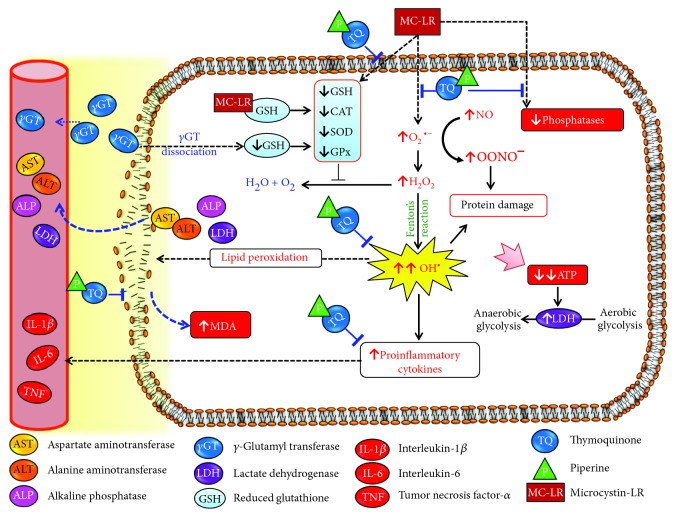
Schematic diagram of the possible antioxidant and anti-inflammatory effects of thymoquinone (TQ), piperine (PP), and their combination against microcystin- (MC-) LR-induced hepatotoxicity and neurotoxicity in mice.

Interestingly, cotherapy of TQ and PP offers plenty of phenol rings and H^+^ donors by which they could obviously improve all parameters much better than their individual treatments. PP is described as a dietary enhancer used to increase the bioavailability of other antioxidants either by promoting their absorption or by slowing their biodegradation in the liver via inhibition of several cytochrome P450-mediated metabolic pathways [53]. PP has been reported to enhance the bioavailability of ginsenoside Rh2 [54], puerarin [22], and (-)-epigallocatechin-3-gallate [23]. Talib (2017) has documented a synergistic action between TQ and PP against breast cancer in a mouse model. Therefore, we assume that the enhanced protective effects of the combined treatment were likely due to the synergistic antioxidant capacity of both agents and the increased bioavailability of TQ.  Figure 5 shows the possible antioxidant and anti-inflammatory effects of TQ, PP, and their combination against MC-induced hepatotoxicity and neurotoxicity.

## 5. Conclusion

In the current study, MC provokes hepatotoxic and neurotoxic effects indicated by elevated serum hepatic markers and proinflammatory cytokines along with disruption of the oxidative state. An electron transport chain may be a possible target for MC. Treatment with TQ or PP alone alleviates such injuries in MC-intoxicated animals which may be attributed to their antioxidant and anti-inflammatory properties. Notably, a combination of TQ and PP exerts more improvement than their individual treatments. Such efficacy might be due to providing more than one antioxidant and due to the PP-enhanced bioavailability of TQ.

## Data Availability

The data used to support the findings of this study are available from the corresponding author upon request.

## Abbreviations

ALT: Alanine aminotransferase
ALP: Alkaline phosphatase
AST: Aspartate aminotransferase
CAT: Catalase
*γ*GT: Gamma-glutamyl transferase
GSH: Reduced glutathione
GSH-Px: Glutathione peroxidase
IL-1*β*: Interleukin-1*β*
IL-6: Interleukin-6
LDH: Lactate dehydrogenase
MDA: Malondialdehyde
SOD: Superoxide dismutase
TNF-*α*: Tumor necrosis factor-*α*
TQ: Thymoquinone
MC-LR: Microcystin-LR.
